# The Meaning of Life, Equality and Eternity

**DOI:** 10.1007/s10892-019-09296-0

**Published:** 2019-06-21

**Authors:** Ingmar Persson, Julian Savulescu

**Affiliations:** 1 Göteborg University, Göteborg, Sweden; 2 The Oxford Uehiro Centre for Practical Ethics, University of Oxford, Suite 8, Littlegate House, St Ebbe’s Street, Oxford, OX1 1PT UK; 3 Wellcome Centre for Ethics and Humanities, University of Oxford, Oxford, UK; 4 Murdoch Children’s Research Institute, Melbourne, Australia; 5 Melbourne Law School, Melbourne, Australia

**Keywords:** Meaning of life, Value of life, Equality of, From Eternity, Susan Wolf

## Abstract

We present an analysis of a notion of the meaning of life, according to which our lives have meaning if we spend them intentionally producing what has value for ourselves or others. In this sense our lives can have meaning even if a science-inspired view of the world is correct, and they are only transient phenomena in a vast universe. Our lives are more or less meaningful in this sense due to the difference in value for ourselves and others we intentionally create while leading them. These inequalities are morally unjustifiable because they are ultimately due to factors beyond our responsibility and control. But from the point of view of eternity these differences in meaningfulness and value dwindle to insignificance, and this offers some consolation for the unjustifiable inequalities.

## Introduction

While human beings have puzzled over the meaning of their lives for thousands of years, the issue became more challenging in the Western world as Christianity lost ground to modern science and philosophy. According to a traditional Christian view of the world, our lives are embedded in a divine scheme in which they are mere preparation for an eternal afterlife. A human being is *Imago Dei*, made in the image of the creator of the universe, and the Earth is taken to be the centre of the universe. According to modern science and philosophy, we are instead as mortal as the non-human animals from which we have descended and with which we share the world, and this planet is a vanishingly small, perishable speck in an infinite universe. Eventually, this planet will be gone without a trace.

We are social beings with an almost uncontrollable predilection for explaining things in mental and moral terms. This predilection is useful in our dealings with other human beings whose behaviour is indeed explainable in such terms. But it is so powerful that it produces false positives to the effect that events can be given mental or moral explanations when in fact, as science has convincingly shown, this is not so. In early human societies, *animism* was prevalent, that is, processes in inanimate nature were given mental explanations. Thus, a crop failure was seen as the result of the anger of some supernatural agent, the behaviour of liquids was explained by reference to their *horror vacui*, and so on. Such explanations not only make these processes comprehensible, they also promise us a possibility of control, e.g. that we could prevent future crop failures by appeasing the angry supernatural agent with suitable sacrifices. Science has however long since made such animistic explanations obsolete by supplying highly successful mechanistic explanations.

Nonetheless, in modern societies our disposition to supply mental explanations still works overtime, though less blatantly than in animistic societies. People are still inclined to think that, for instance, misfortunes signify something, have a meaning. They ask questions like ‘Why am I so unlucky, and Richie so lucky?’ as though they expected a moral reason justifying this. They are spontaneously inclined to attribute mental states to dead human beings—witness how common it is for people who are mourning to try to talk to the deceased and ask for forgiveness, etc. It has been suggested that this wellnigh irresistible urge to seek explanations in intentional and moral terms may account for why belief in afterlife alongside religious beliefs in supernatural powers are found in societies all over the world at all times (see e.g. Boyer 2001).

When people are forced to spend most of their time doing boring work in order to earn their livelihood, they might find their lives meaningless *in comparison to* the lives of people who are fortunate enough to be in a position to spend a lot of their time on interesting or pleasant pursuits. But when many people ask the general question whether *any* human life can have meaning, we suggest that what they ask for is what role or purpose their lives have in a cosmic plan or drama. This is however a question that presupposes that there is some superhuman intelligence devising such a cosmic plan or drama. On a scientific and secular view, our lives can have no meaning in this sense.

According to science, planet Earth is indeed a vanishingly small speck in a huge universe. The conclusion is inescapable that whatever we do, or whatever happens to us, will have virtually no impact on this universe (cf. Nagel 1986: 11.2). We are oblivious to this cosmic perspective while we engage in the pursuits of everyday life, but when we sit back and contemplate our lives *sub specie aeternitatis*, this vast vista opens up and makes our lives appear transient and futile. However successful we are in our undertakings, however fulfilled and influential they make us, we along with all our achievements are ephemeral on a cosmic time-scale; eventually all traces of us will be erased. Thus, it seems that from this detached point of view from above our lives cannot but be meaningless. We can suppress this insight by indulging headlong in what our earthly lives have to offer but, if we are reflective enough, it will now and then creep in on us, and a sense of meaningless is prone to take possession of us. Our ordinary state of mind with all its anxieties and pleasures will then seem like a state of intoxication from which we are sobering up to a cold and bleak reality.

There is a considerable amount of truth in these reflections in light of the cosmic picture, but they are exaggerated. They tend to make the mistaken assumption that the meaning of life is an all-or-nothing matter: either life has meaning or it is meaningless. We instead take up the common suggestion (see e.g. Metz 2013: 4) that there are *degrees of meaning*: human lives can have more or less meaning. The meaning of life is a scalar notion.

However, the fact that some human lives are more meaningful than others raises another problem. This is because some people’s lives are often less meaningful than the lives of other people through no fault or voluntary choice of these people. For instance, some people are born with serious genetic diseases that significantly limit their mental and physical capacities and life-span. Simply through bad luck, others fall victims to grave accidents early in life. It seems that it cannot be just that these people lead less meaningful lives than people who are born healthy and go through long and happy lives without being exposed to incapacitating accidents. Nor can it be justified for any other moral reason, such as its utility. To some extent, we might be able to rectify the moral unjustifiability of these inequalities by making societies safer and more equal and by genetically enhancing hereditary human capacities. The progress of science and social engineering is putting in our hands increasingly powerful means to these ends. Nevertheless, they will surely never make us almighty (and perfectly moral in the administration of our power), so a lot of unjustifiable inequality in respect of welfare or well-being is bound to remain.

The cosmic perspective which threatens to be wholly destructive of the meaning of human life can then be seen to have a redeeming aspect. Against a boundless eternal backdrop, it will appear that even the most successful human beings achieve comparatively little. Even the most lasting and profound achievements shrink to insignificance in a cosmos which is infinite in space and time. Thus, although it remains true that some human lives are more meaningful than others, the difference in meaning will shrink to next to nothing against the backdrop of a cosmic setting; eternity will almost equalize the differences in meaning between human lives. So, the unjustifiable inequality in respect of meaningfulness will be less glaring, though it will still exist, and the remaining small differences are not a matter of indifference.

From the more engaged, personal perspective that we adopt when we conduct our everyday life, these differences appear by contrast to be of first-order importance. If our frame of reference allows for rather limited variations in respect of some good, such as income, it only takes our neighbours to earn slightly more than us for us to be envious of them, whereas if our frame of reference allows for greater variation as regards income by including the richest people, nationally or internationally, it takes much more to arouse our envy. We do not react just to the absolute size of the income difference between us and someone else, but to the size of this difference relative to a background of differences that is our frame of reference.

## The Meaning of ‘The Meaning of Life’

It is useful to draw a broad distinction between two meanings of ‘life’: ‘life’ in a *biological* and a *biographical* sense.^1^ Our biological life consists in our organisms being involved in processes like breathing, circulating blood, digesting food, and so on. Our biographical life rather consists in the sum of events which happen while we are biologically alive and which make some difference to how we act and react, like the events that we would write about were we to write our autobiography. Possession of consciousness is necessary for having a biographical life, but not for having a biological life, e.g. plants can be biologically alive, but they certainly do not have life in the biographical sense. However, possession of the most rudimentary kind of consciousness—for instance, the kind of consciousness insects are endowed with—is not sufficient for having life in a biographical sense if this requires what we could properly speak of someone as ‘leading’ a life. This takes agents who are capable of being conscious of the ends they have, and of judging what means are suitable to achieving them.

Dennett (1978: 65) describes a wasp whose routine it is to drag a paralyzed cricket to the threshold of its burrow, go inside to check that all is well, and then drag the cricket into the burrow. But if the cricket is moved a few inches while the wasp is inside the burrow, it will repeat the procedure of dragging it to the threshold and going inside to check. It can be made to repeat this routine an endless number of times. This is because wasp does not understand the meaning or point of dragging the cricket to the threshold and checking the burrow just before dragging it inside. Evolution has equipped the wasp with an instinct to this effect, presumably because it serves its reproductive fitness. But the wasp does not understand what the purpose of its behaviour is, and cannot modify its behaviour in circumstances in which it no longer serve its purpose. It is not self-conscious to the extent that it is capable of making the evolutionary end of reproductive fitness *its own* end, something to which it attaches value, and to look for effective means to attain it. This is necessary, we claim, for these means to have meaning *for it*, *personal* meaning.

The lives of individuals can have such meaning only if they lead (biographical) lives which contain actions and attitudes that have such meaning. In the actual world, these activities must include the activity of keeping themselves biologically alive because individuals cannot conduct any other activities that are meaningful to them unless they have consciousness for which biological life is necessary. This is so because we suggest that an activity that you engage in could have meaning for you only if it *intentionally* produces some good. The personal meaning your life can have is then dependent on your capacity to have intentions.

Suppose that you sit idly in a coffee shop, just whiling away your time, but that unbeknownst to you, your presence scares off a robber who would otherwise have held up the shop. Then your sitting in the coffee shop unintentionally produces some good, but we would not say that your sitting there had meaning for you if you felt that sitting there was a waste of time. You have to mean or intend to produce the good that your activity in fact produces, and this is not so in this case.

By contrast, Aaron Smuts argues that ‘the good effects that count towards the meaning of one’s life need not be intentional…What matters is that one is causally responsible for the good’ (2013: 536). But suppose a terrorist has placed a powerful bomb in a stadium with 100,000 spectators. It is set to go off in one hour, killing all the spectators. However, a snail crawls into the bomb, is crushed by a mechanism in it, and thereby causes the bomb to jam. The snail is causally responsible for or the cause of the good effect of saving all those people, but surely this is not enough for its life to have meaning for it. In fact, life cannot have meaning for a simple creature like a snail.^2^ We maintain that this is because it is incapable of leading a life in the biographical sense, and this is because it cannot *intentionally* produce good effects. Nonetheless, the snail’s life can have meaning *for one of us* who intentionally places it where it will crawl into the bomb and prevent its detonating.

Bramble (2015) advances a view which is exposed to the same kind of objection as Smuts’ view. Bramble is however interested not in our lives having meaning *for us*, but in their having meaning. However, it seems to us that *anything*—including inanimate objects—that could effectively be used for a valuable end could be said to have meaning (for users, we would add). But meaning in this broad sense is surely not what we have in mind when we wonder about the meaning of our lives.

Returning to human agents, imagine instead that your action fails to produce the good that you intend, and produces no good whatsoever, e.g. you intend to rescue somebody, but fail to do so and achieve nothing of value. Then your action is meaningless, a waste of your time and energy. This is precisely why Sisyphus’ attempt to roll the boulder up a hill in a famous myth of ancient Greece is seen as a paradigm instance of a meaningless activity; he fails to place the boulder on the top because it rolls back all the time.

However, the condition of an activity intentionally producing goodness cannot be sufficient for it to be meaningful. For an activity might bring about the good effects intended, but in addition intended or unintended bad effects that outweigh them.^3^ We would then be disinclined to say that the activity had meaning. So, to obtain a condition which is both necessary and sufficient for the meaningfulness of a life, we have to maintain something like this: your life can have personal meaning only if you spend your life intentionally producing *a net balance of goodness*.

We should also distinguish between what is good *for you* and good *for others*. Imagine you spend your life intentionally doing things that have value only for yourself, e.g. that give yourself pleasure, but that you produce nothing of value for others. Taylor (1981) claims that such a life is meaningful: he conducts the thought-experiment of imagining that Sisyphus is enjoying (rather than enduring) rolling the boulder up the hill more than he enjoys anything else, though it always rolls back. Taylor claims that Sisyphus’ life would then have meaning, even though it does not result in anything that is valuable for anyone else.

Susan Wolf rejects Taylor’s view because, in spite of his enjoyment, Sisyphus’ activity ‘remains futile’ (2010: 17).^4^ She claims that in order to be meaningful, apart from being subjectively fulfilling, a life must contribute to ‘something the value of which is (in part) independent of oneself’ (2010: 22). According to her, ‘a life is meaningful insofar as its subjective attractions are to things or goals that are objectively worthwhile’ (2010: 34–35). She confesses that she has ‘no positive account of nonsubjective value with which I am satisfied’ (2010: 45). But she leans towards an intersubjectivist account of objective or nonsubjective value according to which ‘almost anything that a significant number of people have *taken* to be valuable over a long span of time is valuable’ (2010: 47). We want however to insist that our lives can have personal meaning *for us*, though they produce something that is of value only to ourselves. The reason for this surfaces in something Wolf herself observes:

there seems good reason to ask why, if an activity’s value to *oneself* is insufficient to give meaning to one’s life, an activity’s value to some *other* creature should make it any more suitable. (2010: 38) It may seem odd that if I benefit you and you benefit me, our activities may contribute to the meaningfulness of each other’s lives, but if we each tend to our own well-being, our actions will have no such effect. (2010: 42)

Since we cannot see that Wolf has any satisfactory answer to this kind of query, we take it that spending our lives intentionally promoting what is of value to ourselves is sufficient to provide it with personal meaning for ourselves. Spending it intentionally promoting what is of value only to others would also make our lives meaningful. This situation is however barely conceivable, for if we intentionally satisfy our desire to provide what has value for others, we are likely to be aware of this fact to some extent, and feel the pleasure of satisfaction. But our feeling pleasure is probably something we desire; so we also accomplish something that is good for ourselves. In principle, it is however possible for us to lead a life that has personal meaning only in virtue of the value for others it intentionally creates, for example, if we mistakenly believe that our actions have failed to promote the intended value to others. On the other hand, if we spend our lives trying to create what is good only for others, but fail to do so, while being grossly deceived and believing that we have succeeded, our lives could have meaning for us in virtue of the pleasurable satisfaction they contain as the result of our false beliefs.

What about the lives of satanically wicked people who intentionally cause a lot of misery for others? It is possible to distinguish between *good and bad meaning*, depending on whether the relevant intentions are good or bad, i.e. are directed at goodness or badness for beings (though what we ordinarily mean by the meaning of life is good meaning). This distinction enables us to say that if wicked people intentionally cause some goodness for themselves, but more badness for others, their lives have a personal meaning which is (predominantly) bad.

It should be emphasized, however, that although we are prepared to distinguish between good and bad meaning, we take meaning and value to be distinct notions.^5^ Just like the meaning of what we say is plausibly understood in terms of what we intentionally convey, the meaning of our lives should be understood in terms of what we intentionally bring about in the way of outcomes of (positive or negative) value for ourselves or others. Thus, it is intentional means that have (personal) meaning, whilst it is their ends that have value (for ourselves or others).^6^

In this connection, it should be pointed out that we conceive the notion of being of value to somebody more broadly than does Wolf. She takes it to imply ‘a form of hedonism’, according to which pleasure and other mental states alone are of value (2010: 15). But consider someone who spends his life trying to achieve something that will make him remembered for thousands of years, long after his death, and succeeds. Even if this achievement is something that does not have (positive) value for others, this success is enough to give his life some meaning for him, by fulfilling a leading aim or desire that this person had in his life. This is so, though he is no longer around to enjoy or feel pleased by the successful fulfilment of the dominant ambition of his life, since he is long since dead. Of course, hedonists deny this. Thus hedonism, even if it is a part of the correct account of value for us, does not fully constitute it. A life like Herostratus’—who burnt down the Temple of Artemis to gain immortal fame, knowing he would be executed—can have meaning for him. For what is needed for our life to have meaning for us is that our actions *in fact* fulfil some desires of ours in such a way that we intentionally realize some desired value, not that we are *aware* of this fulfilment and feel satisfaction, as hedonists would insist on.

The value that gives personal meaning to life, then, consists in the intentional fulfilment of desires: value for ourselves if it is the fulfilment of our desires that we ourselves obtain something, and value for others if it is the fulfilment of their desires that they obtain something. But many affirm that in order for the fulfilment of a desire to be of value the desire must satisfy some *requirement of correctness*. There are different ways of understanding such a requirement. According to one account, a desire is correct and worth satisfying if it is not based on any factual mistakes. This account implies that if you form a desire in light of knowledge of all relevant facts (available), the fulfilment of this desire is of value. According to a stronger account, there are additional, objective norms of conative correctness that a desire must pass in order to be correct. Then even if a desire is formed in light of knowledge of all relevant (available) facts, the fulfilment of it might not be of value.

This highly controversial issue cannot be resolved here, but we must confess that we do not see how to construe an objective standard of which it could plausibly be claimed that it must be satisfied for anything to be valuable *for individuals*.^7^ It is harder to believe that objectivity is necessary for something to be valuable for individuals than for something to be valuable full stop. For instance, it is hard to believe that whether it is bad for you to experience a pain you strongly dislike hinges on whether there is some objective standard that your aversion to this pain must meet, since you can rationally be *certain* that this pain is bad for you, but not that there is such a standard.^8^

We have distinguished between the personal meaning your life can have for you in terms of what your activities intentionally produce that is of value to you, and the personal meaning your life can have for others in terms of your intentional contributions to what is of value to others. The meaning of a life for both reasons is a matter of degree. Naturally, differences in the degrees of the meaning of a life in virtue of the value for others it can intentionally produce are much greater than the differences in the degree of the meaning it can have in virtue of the value it intentionally produces for the person leading the life, since the surplus of the good for others that agents can intentionally produce can be so much greater than the good for themselves that they can intentionally produce.

This distinction between degrees of meaning enables us to deal with some imaginary cases that Wolf describes. She writes of a woman ‘whose life revolves around her pet goldfish’ that, though perhaps ‘the life and comfort of a goldfish is worth *something*’, these things ‘do not seem valuable *enough* to merit the kind of time, energy, and investment’ that the woman devotes to them, particularly not in light of the wealth of other things that she could devote herself to (2010: 37–38). But this does not seem to us to be a reason for denying that focusing on the well-being of a goldfish can provide the life of the woman with *some* personal meaning. It seems more natural to claim that it can provide it with only very little meaning compared to other things to which the woman could have devoted herself. It would seem that if the woman had devoted herself to the well-being of *a whole lot* of animals, Wolf would have to concede that this could make her life meaningful, but there is only a *difference of degree* between this case and the case of concern for a single goldfish. Therefore, we conclude that it is more natural to claim that this woman’s life has very little meaning than no meaning whatsoever.

## The Moral Unjustifiability of Lives Being More or Less Valuable and Meaningful

Obviously, the lives of people differ radically with regard to their contributions to the value of the lives of their fellow-beings. Think for instance of people who have created artistic masterpieces, like Leonardo da Vinci, William Shakespeare and Wolfgang Mozart, people who have made great scientific discoveries, like Isaac Newton, Albert Einstein and Alexander Fleming, or people who have founded world-wide religions, like Buddha, Confucius, Jesus and Muhammad. Since the achievements of such people have had—at least in part intentionally—an impact on the lives of others for centuries, they have contributed to the good of others to an extent that enormously exceeds the impact of the lives of more ordinary people.^9^ Thus, they had lives that are vastly more meaningful than average people have. But it is clear as well that humans also vary considerably in respect of the value they could inject into their own lives. Some people fail to inject much value into their own lives because they are lazy or destitute, others because they are mentally or physically incapable.

To a great extent, the fact that the lives of some humans have less (good) meaning than the lives of many others is not due to the fault or voluntary choice of these people. They are struck down by some misfortune or other. Many people will contribute less to the value of their own lives and the lives of others because they happen to be born into social conditions which leave them malnourished, unhealthy or uneducated. Others are genetically disadvantaged and suffer from severe mental or physical congenital impairments. Still others who are genetically and socially well-endowed from the start have their lives stunted by unforeseen accidents, crimes or diseases which kill or cripple them prematurely. Through no fault or voluntary choice of their own, these individuals lead lives that are less meaningful than the lives of other, more fortunate individuals because they produce less value for themselves or others.

To our minds, it sounds plausible to claim that when the lives of some humans are less meaningful through no fault or voluntary choice of their own, this is *unjust (or unfair)*. But some theorists want to restrict the application of the terms ‘just’ and ‘unjust’ to the outcomes of acts of morally responsible agents. These theorists should however agree that inequalities in respect of the value and meaningfulness which are due to natural forces are *not just*. For this means that these inequalities are *not* in accordance with justice, and this could be because considerations of justice do not apply; it need not be because they are unjust, i.e. contrary to justice. It is sufficient for our purposes that these inequalities are not just. Since they are not morally justifiable for other reasons—in particular the utilitarian one that they are conducive to a greater sum of good overall—they are *morally unjustifiable*.

To some extent, these inequalities can be rectified by human action. We can improve the socio-economic conditions of the worse-off, so that they will be better nourished and educated and, hence, better equipped to lead lives of value to themselves and others. We are also beginning to acquire gene therapies and other biological interventions (such as stem cell therapies, bionic body parts or exoskeletons) to cure or mitigate some congenital diseases and disabilities, so that not only the socio-economic factors, but also the genetic or biological start of human lives could become more equal. Science and medicine constantly make progress that enables us to cure or alleviate more diseases. The fight against violent crimes can be made more effective, roads more secure, so that fewer people fall victims to violent crimes and traffic accidents, and so on.

But it is unlikely that we shall ever succeed in equalizing all of the unjustifiable inequalities in respect of the value and meaningfulness of lives. Some socio-economic differences will remain that will help some to a better start than others. So will some genetic disadvantages, and there will be some unforeseen accidents, crimes and diseases which claim or stunt lives prematurely. It is most regrettable that such unjustifiable inequalities will remain, though their existence by no means implies that all people who are disabled, poor or die prematurely necessarily lead lives that have less meaning for them than the lives of people who are not (as) disabled or poor, or die (as) early. Nor does it imply that these less fortunate people cannot lead lives that are very good for them and others and, thus, have a great deal of (good) meaning. Furthermore, changes in respect of the prejudicial attitudes to these people can accomplish a lot of improvement.^10^

At this juncture, it is worth saying something about how the notion of your life being less meaningful through what you choose to do with it is to be understood, and to reflect on another common everyday dilemma in connection with the meaning of life. When considering how to live your life, you might well ask yourself whether you should ‘live for the moment’ or pursue some more long-term goals, such as writing a book or working for some political cause, though this requires you to sacrifice some immediate rewards. It might well be that, if you succeed in attaining the long-term goals, your life will be more valuable both to yourself and others than it would be were you successfully to live for the present moment. But if you fail in attaining the long-term goals—perhaps because some unforeseen accident, crime or disease prematurely kills or incapacitates you—it will be less valuable in both respects. Imagine that you choose to spend your life pursuing the long-term goal, but fail to attain it due to some fatality that you could not possibly have foreseen. Then your life is rendered less meaningful in some sense because of your voluntary choice. However, this is not the sense which removes the moral unjustifiability of your life being less meaningful, since you do not choose to lead a less meaningful life, but to pursue a long-term goal that eludes you. Your leading a less meaningful life is not intentional or foreseen, but accidental.

Clearly, this dilemma of having to choose between living for the moment or living for long-term goals is inescapable so long as we cannot reliably predict what the outcomes of our choices will be. Presumably, we shall never be able to predict this in any detailed way. Moreover, in the unlikely event that we were to be capable of making such detailed predictions, a lot of the point of living would be lost, since much of this point concerns finding out what we are capable of achieving and what it is like to lead a life in possession of these capacities (cf. Persson 2005: 148–150).

## The ‘Equalizing’ Effect of Eternity

We have defended a view according to which our lives can have (personal) meaning, to a greater or lesser degree, on a scientific and non-religious understanding of the universe. Our lives can have (such) meaning due to the value we intentionally produce for ourselves or others, even though death is the end, and there is no eternal afterlife of the sort that many religions postulate. Now it is certainly good news that our lives are not necessarily meaningless according to a scientific picture of the universe, as many religious believers and atheists alike have feared. But our view also states that some of us lead (much) more meaningful lives than others and that this is often morally unjustifiable. Since morally unjustifiable inequalities are bad, our view also carries bad news (especially as some of these inequalities are inescapable). In respect of justifiability, the nihilist view that all human lives are meaningless is more appealing than the view here defended, since it does not imply that there is any unjustifiable inequality in respect of meaningfulness—though it establishes equality by means of a radical devaluation of our lives, by the draconic measure of depriving all lives of meaning. We might ask whether our view could acquire something of the egalitarian merits of the devaluative view without this drawback.

Let us look at one of the most famous accounts of the experience of life as meaningless, namely Leo Tolstoy’s. At the age of about fifty, Tolstoy was seized by a feeling that his life was meaningless, though he ‘was on every side surrounded by what was considered to be complete happiness’ (1981: 10): he was a famous writer, a rich land-owner, and had a devoted wife and a large family. The origin of Tolstoy’s feeling of meaninglessness seems to have been an awareness that none of all this fortune and happiness would last:

Sooner or later there would come diseases and death… to my dear ones and to me, and there would be nothing left but stench and worms. All my affairs, no matter what they might be, would sooner or later be forgotten, and I myself should not exist. (1981: 11)

It appears that Tolstoy was of the opinion that his life could have meaning only if there were something eternal and indestructible about it:

The question was ‘Why should I live?’ that is, ‘What real, indestructible essence will come from my phantasmal, destructible life?’ (1981: 15) ‘What is the meaning which is not destroyed by death?’ – ‘The union with infinite God, paradise’. (1981: 16)

The fact that life is nothing but ‘a particle of the infinite not only gives it no meaning, but even destroys every possible meaning’ (1981: 14). In sum, Tolstoy’s view seems to be that if our lives are to have meaning, they must go on forever, in a way that is overall valuable, or at least they must result in something of eternal value. If this is right, a scientific, secular view of the universe will imply that our lives are meaningless because our death will be tantamount to our annihilation. Consequently, whatever value we bring to our lives or the lives of others will fade gradually to nothing in the course of eternity. Our lives being meaningful, according to people like Tolstoy, requires a religious view, like the Christian, which offers the prospect of an eternal paradise after this life.

However, it is certainly false that something cannot be of value unless it lasts forever, or is of infinite duration. That something is of infinite temporal extension is as little necessary for it to be valuable as it is that it is of infinite spatial extension. Perhaps something cannot be of *infinite* value, unless it is of infinite duration or infinite spatial extension. But why claim that our lives must result in something of infinite value in order for them to be (personally) meaningful; why is it not enough that they (intentionally) result in something of finite value for us or others? Once we distinguish between degrees of meaning, it should readily be seen that in order to have some degree of meaning, it is enough if our lives intentionally result in something of finite value for ourselves or others. Of course, our lives would be vastly more meaningful if they resulted in something of infinite value to ourselves or others, but that is no reason to deny that their resulting in something of finite value is capable of supplying them with some meaning.

But when you adopt a cosmic perspective which opens up a universe that is apparently endless both spatially and temporally, why is it so tempting to deny, as Tolstoy apparently did, that anything that we could do here and now on Earth could make our lives meaningful? This must doubtless be tempting, since he is far from being the only one who has succumbed to this temptation.^11^ If you view a valuable everyday state of affairs, such as your winning a large sum of money, from a mundane personal perspective which often does not range over more than our neighbourhood and the near future, this state of affairs could occupy a relatively large part of your perspective. For this perspective cannot harbour states of affairs that are hugely more extensive in space and time. But with a switch to a cosmic perspective which ranges over more of the universe than the Earth, and even of the galaxy to which it belongs, and over billions of years, vastly more extensive states of affairs become imaginable. In comparison to them, what we could accomplish in our lives dwindles to something so small that we may find it difficult to care about it. If we take into consideration the billions of years during which we shall be dead, a few decades of happiness, say, before we die is likely to seem too insignificant to bother about. By contrast, if our time frame does not stretch beyond the rest of our lives, and we compare being happy with being unhappy during those decades, it comes out as being definitely better to be happy, and we shall be keen to be happy rather than unhappy. The loss of concern about a few possible decades of happiness that we experience when we shift from this mundane perspective to an immensely more extensive cosmic perspective is so drastic that it might appear to us that this period of happiness loses *all* value, though this is strictly speaking not true.^12^ This might explain why people who ascend to this point of view, like Tolstoy, come to perceive life as meaningless, despite the fact that this is an erroneous exaggeration.

Although adopting the cosmic point of view can involve such a negative exaggeration, it must not be confused with situations in which we claim that our life is meaningless because we take a rashly selective view of it. Consider people who spend most of their life in pursuit of some aim—perhaps they aim to create a great work of art, to make some important scientific discovery, or to promote some political cause—and in old age find out that they have failed to achieve this aim. They might then feel that their entire life has been meaningless, a waste of time and effort. At this moment of disappointment, they are prone to overlook that they have spent many long periods of their lives happily engrossed in the pursuit of this goal, experiencing what has been called *flow* by the psychologist Csikszentmihalyi (1990). They might also overlook the joy they have brought to their family and friends.

In general, it is exceedingly difficult to make a balanced estimate of the good and bad you have done to yourself and others during your life-span. You are prone to be guided by some episodes in your life that readily present themselves to you, e.g. what you experience right now.^13^ Such misguided estimates could be self-fulfilling: if you judge that your life has been going badly, this might cause you to make your life take a turn for the worse. On the other hand, misguided positive estimates are likely to be self-fulfilling: if you judge that your life has been going well, this might make your life go better than it otherwise would have gone. But notice that we are more likely to make misguided negative estimates because we are more inclined to reflect on our lives when we are dejected and bored than when we are fulfilled and stimulated. In the latter mood we simply immerse ourselves in the activities of life, enthusiastically getting on with the business of living.

You are not guilty of such erroneous, partial judgements of your life when you adopt a cosmic perspective: this perspective could take into account every fact about your life that the most accurate local personal perspective on your life could take into account. But it covers more by widening the earth-bound context of your life to a cosmic context. In virtue of being more encompassing, the cosmic point of view can claim to present your life in a truer or more rational light than any more mundane point of view it contains. This does not imply, however, that you *should* adopt a cosmic perspective rather than a mundane personal perspective, since you may reasonably dispute whether being more truthful and rational is worth the cost in respect of loss of involvement in life. This involvement is probably necessary to motivate us to make our lives as meaningful as possible to ourselves and others, by promoting what is of value in our own life and in the lives of others. Additionally, a local point of view is probably necessary to motivate us to try to rectify morally unjustifiable inequalities in respect of the value of lives as far as this is possible.

However, to a considerable extent the unjustifiability of some humans leading less meaningful lives than others through no fault or voluntary choice of their own cannot be abolished by us. In so far as this is so, the loss of involvement and concern that the adoption of a universal perspective brings along could provide consolation, by alleviating some of the sting of the feeling of the unavoidable moral unjustifiability in society and nature. Even the achievements of the most influential people, the people whose achievements have affected the history of the world even for millennia, like Plato, Buddha and Confucius, will shrink to minuteness from a point of view which ranges widely over the universe for billions of years. Thus, the cosmic perspective has something of an equalizing effect: its spatio-temporal vastness will greatly diminish the relative differences between more and less meaningful human lives. But, again, this perspective does not completely obliterate the differences in meaningfulness between human lives: it is still true—and important—that some lives are more meaningful than others.

Williams (1973) speculates that if we were to live forever, we would eventually be overcome by boredom. If he is right, eternal life would be terrible, since there would be no possible escape from the boredom of an eternal life (at least if we cannot make ourselves unconscious forever). But it is hard to tell whether he is right, since it is so difficult to imagine a life that goes on forever. To be sure, we can imagine a life that goes on apparently without end, i.e. a life that is such that at whatever point in time we consider it, it stretches beyond this point. There seems to be no reason why such a life cannot be happy and fulfilling, however. The world or universe is seemingly inexhaustible, so alert and curious people could constantly discover new sources of interest. Besides, they might now and then ‘refresh’ themselves by inducing partial amnesia. Compared to such a life lasting for thousands and even millions of years, the few decades of happiness that we could hope for appear trivial. Since such an indefinitely long life is a possible object of comparison *sub specie aeternitatis*, the periods of happiness possible for humans now could appear trivial to us.

If the explanation of the meaninglessness of life is a shift from a mundane to a cosmic perspective, we can understand Ludwig Wittgenstein’s view: ‘The solution of the problem of life is seen in the vanishing of the problem’ and that this is the reason why those who, after doubting ‘the sense of life’, have resolved their doubts have ‘been unable to say what constituted that sense’ (1963: 6.521). If you cease feeling that life is meaningless because you are sucked back into an engaged mundane perspective from a disenchanting cosmic perspective, this feeling could evaporate even though you have not made any new discovery about life that you could articulate verbally. However, the same inability to report a ‘sense’ could also result when your judgement that your life is meaningless has been prompted simply by temporary depression: when this period becomes temporally distant and you enter a happier phase, your gloomy judgement is likely to vanish without a trace.

## Conclusion

We have tried to show that a science-based, secular view of the world is compatible with our lives having meaning if we understand this notion aright. Our lives have (good) personal meaning if we intentionally bring about a surplus of goodness for ourselves or others. Meaning of life in this sense is scalar: some lives are more meaningful than others. It is true that science undercuts the possibility that our lives have meaning in the sense of having some role or purpose in a cosmic plan or drama designed by a supernatural intelligence, for it rolls out a boundless universe in which human affairs occupy only a vanishingly small place. On the other hand, science supplies us with more effective means than ever to supply our own lives and the lives of others with value and to reduce morally unjustifiable inequality in respect of personal meaning and value between human lives. Although the value our lives can have appears minuscule from the cosmic perspective that science opens up, it is a mistake to feel that they have no value and meaning at all. It is a mistake, committed by Tolstoy and others who have been taken in by religious world-views, to think that our lives can have meaning only if they last forever, or make contributions to something that lasts forever. Such a mistake could be self-fulfilling, by draining us of motivation to make our lives as good and meaningful as possible.

## Footnotes

1 We borrow this distinction from Rachels (1986: 5–6, 24–27).

2 In response to a similar objection, Smuts appears prepared to bite the bullet (2013: 558), but he does not consider animals as simple as snails.

3 Bramble’s *Consequentialism about Meaning in Life* seems exposed to this sort of objection because one of its claims is that ‘one’s life is meaningful at *t* just in case one’s surviving at *t* would be good *in some way’* (2015: 445, our italics) rather than good *all things considered*.

4 This is a variant of a standard objection which many have launched against Taylor’s sort of position, as noted e.g. by Metz (2013: 175).

5 Contrast Smuts who takes meaning(fulness) to be ‘a certain kind of value’ (2013: 538) when he alludes to the idea of ‘anti-meaning’ or ‘negative meaning’. Cf. also Metz (2013: 64). There is an inventory of conceptions of anti-meaning in Campbell & Nyholm (2015), but it does not feature the present proposal.

6 Cf. Metz who takes meaning to depend on ‘action under one’s control’ (2013: 69), though he has a more lofty conception than us of the value actions must bring about: they must go ‘beyond one’s own pleasure’ and achieve ‘transcendence of one’s animal nature, and greatly estimable or admirable conditions’ (2013: 60).

7 While Wolf has not found any objectivism with which she is satisfied (2010: 45–47). Metz champions a kind of naturalist objectivism, but even if right, it shows only ‘that objective value is possible… not …that is actual’ (2013: 172).

8 For an elaboration of this argument, see Persson (2017: ch. 1).

9 Of course, we have only the vaguest ideas about the amount of goodness these illustrious people have created for others. There are enormous epistemic problems of knowing the extent to which their deeds have affected others, but also serious axiological problems e.g. about balancing qualities and quantities of value against each other, if we eschew simplistic Benthamite hedonism.

10 On the so-called “social construction” of disability, see Kahane and Savulescu (2009).

11 For recent examples, see Nagel (1986: 11.2) and Nozick’s speculations about the meaning of life culminating in the unlimited *Ein Sof* (1981: 600–610).

12 Cf. the discussion in Persson (2005: 224–227).

13 Cf. Kahneman: ‘the score that you quickly assign to your life is determined by a small sample of highly available ideas, not by a careful weighting of the domains of your life’ (2011: 400).
